# Does Scapular and Thoracic Morphology Affect Latarjet Alpha Angle?

**DOI:** 10.3390/jcm14010274

**Published:** 2025-01-06

**Authors:** Taha Kizilkurt, Muhammed Furkan Darilmaz, Furkan Okatar, Ali Ersen

**Affiliations:** Department of Orthopedics and Traumatology, Faculty of Medicine, Istanbul University, Istanbul 34093, Turkey; drtahakizilkurt@gmail.com (T.K.); furkanokatarorto@gmail.com (F.O.); ali_ersen@hotmail.com (A.E.)

**Keywords:** shoulder instability, Latarjet, thorax morphology, alpha angle

## Abstract

**Purpose:** This study aimed to determine the relationship between alpha angle (the angle between the screws and the glenoid) and thoracic diameters in patients undergoing the Latarjet procedure. Defining the relationship between thoracic morphology and alpha angle is aimed at filling the gap in the literature and improving surgical outcomes. **Methods:** This retrospective study analyzed 74 patients who underwent the Latarjet procedure for recurrent anterior shoulder instability between 2022 and 2024. All procedures were performed by the same surgeon using a standardized protocol to ensure consistency of surgical technique across cases. In postoperative chest CT scans, alpha angle, anteroposterior diameter of the thorax, transverse diameter of hemithorax, scapular inclination, and glenoid version were evaluated. **Results:** The study included predominantly male patients (90%) with a mean age of 26.4 ± 6.4 years who underwent Latarjet procedures predominantly on the right side (60%). Significant associations were observed between thoracic morphology and alpha angle on postoperative CT scans. There was a significant positive correlation between anterior-posterior/transverse diameter ratio (AP/T) and alpha angle (r = 0.407, *p* < 0.001), as well as correlations between scapular inclination, glenoid version, thoracoscapular angle, and alpha angle (r = 0.275, *p* = 0.018; r = 0.241, *p* = 0.039; r = −0.288, *p* = 0.013, respectively). Patients were divided based on an alpha angle threshold of 15 degrees, with results indicating worse outcomes for angles above this threshold. Additionally, the AP/T ratio demonstrated predictive value for poor outcomes (AUC = 0.660, *p* = 0.018) with a threshold of 1.2545. **Conclusions:** This study highlights the direct impact of thoracic morphology on the alpha angle observed on post-Latarjet chest CT scans. Specifically, patients with a higher ratio of anterior-posterior to transverse thoracic diameter (AP/T) show a proportional increase in alpha angle. When the AP/T ratio exceeds 1.25, surgeons may face challenges in achieving the target alpha angle.

## 1. Introduction

In cases of recurrent shoulder dislocations with anterior glenoid rim bone loss, the transfer of the coracoid process to the anteroinferior glenoid was described in 1954 by Latarjet [1]. This procedure, commonly referred to as the Latarjet procedure, is known for its efficacy in restoring glenohumeral stability. However, successful results depend on precise graft and screw positioning, with deviations often leading to complications such as recurrence (medial transfer more than 10 mm) or osteoarthritis (lateral placement) [2,3]. The alpha angle, defined as the angle between the screws and the glenoid surface, has emerged as a critical metric influencing the biomechanical stability of the graft [4].

Despite its significance, the variability in alpha angle measurements and the underlying anatomical contributors remain underexplored. Our observations of increased alpha angles in patients with distinct thoracic morphologies prompted this investigation. Thoracic morphology, encompassing parameters such as anterior-posterior (AP) and transverse diameters, has been hypothesized to influence scapular positioning and, subsequently, screw trajectories during surgery. Notably, thoracic dimensions are known to correlate with scapular kinematics, as highlighted in studies on shoulder biomechanics [5,6].

Furthermore, the alpha angle’s clinical implications have been well-documented, with thresholds exceeding 15 degrees associated with suboptimal outcomes [2,4]. However, the existing literature has predominantly focused on surgical techniques, with limited emphasis on patient-specific anatomical predictors. This study aims to address this gap by exploring the relationship between thoracic morphology and alpha angle deviations, with the goal of enhancing preoperative planning and surgical precision.

In this context, we standardized alpha angle measurements using axial CT imaging protocols previously validated in biomechanical studies [6]. The theoretical basis for our hypothesis draws from both clinical and basic science perspectives, suggesting that thoracic morphology could serve as a predictor of intraoperative challenges and postoperative outcomes. By elucidating these relationships, this study seeks to provide actionable insights for optimizing the Latarjet procedure. The alpha angle was chosen as the primary metric because it directly correlates with screw positioning and graft stability, which are critical for surgical success in the Latarjet procedure.

## 2. Material and Methods

Patients who underwent the Latarjet procedure for recurrent anterior shoulder instability between 2022 and 2024 were included in the study. As part of the routine protocol, we perform postoperative CT evaluation to assess graft and screw placement in all patients. Of these patients, those in whom CT evaluation of the hemithorax was possible were included in the study. CT evaluation of the hemithorax was deemed feasible for all selected cases. Exclusion criteria were as follows: Patients with incomplete or suboptimal CT imaging; those with previous shoulder surgeries or structural abnormalities affecting thoracic anatomy; patients with non-standard thoracic morphology or prior trauma that might alter radiological assessments.

Post-Latarjet procedure thoracic CT scans were examined based on hemithorax measurement standards, as described in the radiographic evaluation section. A total of 74 patients meeting the criteria were included in the study. The institutional review board of the Istanbul Medical Faculty of Istanbul University (24 May 2024-2573187) has approved this study. All cases have been performed by the same surgeon using the same surgical procedure, which is addressed under the surgical procedure section.

### 2.1. Surgical Preparation and Procedure

All patients were prepared in the beach chair position under general anesthesia, with the upper extremity freely prepared to allow for movement. The scapular retraction was achieved by placing a supportive pillow between the scapular regions. The back of the operated side was left exposed. After palpating and marking the coracoid process, skin and subcutaneous tissue incisions were made using an anterior axillary approach. Following the opening of the deltopectoral fascia and osteotomy of the coracoid block from its root, provisional drilling was performed using the free-hand technique. Subsequently, a transverse incision was made at the level of the anterior-inferior glenoid rim, opening the capsule and subscapularis muscle. After provisional drilling using the free-hand technique, bone graft transfer to the glenoid anterior was achieved through holes obtained, and fixation was performed using one partial-threaded and one fully threaded 4 mm cannulated screw.

### 2.2. Radiographic Evaluation

In the chest CT scans taken in the first postoperative week (within 7 days post-surgery), the following evaluations were performed for all patients: (1) alpha angle, (2) scapular inclination angle, (3) thoracoscapular angle, (4) glenoid version angle, (5) thoracic anterior-posterior diameter, (6) hemithoracic transverse diameter. All assessments were conducted using the PACS (Picture Archiving and Communication Systems) system. Measurements were taken from axial sections in the chest CT scans. A three-window system, simultaneously displaying sagittal and coronal sections, was employed to standardize the axial section. The measurements were repeated at different times by one orthopedist (a separate observer from the surgical team) and two expert radiologists (musculoskeletal specialists). The intraclass correlation coefficient (ICC) was analyzed to determine the reliability of the measurements.

**Figure 1:**
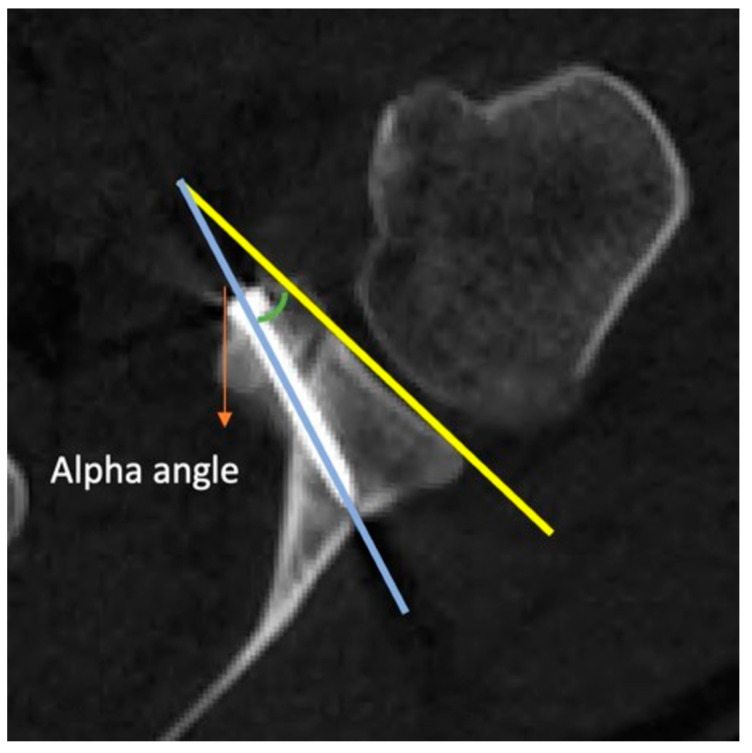
Measurement of the alpha angle on postoperative CT.

Measurement of Alpha Angle: An axial CT image of the glenohumeral joint is captured. The most prominent subcortical points of the anterior and posterior corners of the glenoid are connected. In the axial section, the angle between this line and the body of the screw is referred to as the alpha angle (Figure 1).

**Figure 2:**
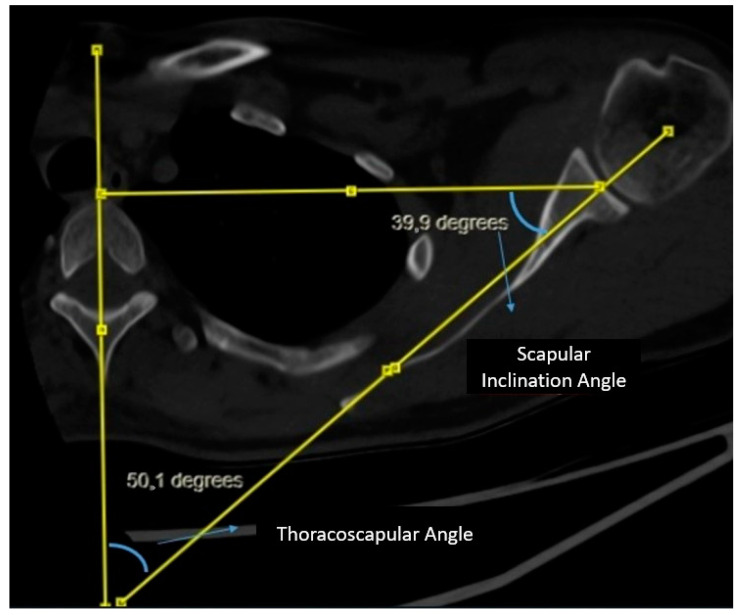
Measurement of scapular inclination and thoracoscapular angle.

Measurement of Scapular Inclination Angle and Thoracoscapular Angle: A vertical line is drawn from the midline of the corpus and spinous process of the thoracic vertebra at the level of the spine of the scapula. Subsequently, a second line connecting the center of the glenoid to the spine of the scapula is drawn, intersecting the line through the midline of the thoracic vertebra. A triangle is formed by drawing a line from the center of the glenoid perpendicular to the vertical line on the vertebra. The angle between the line connecting the center of the glenoid to the spine of the scapula and the line drawn perpendicular from the center of the glenoid to the vertical line on the vertebra is evaluated as the scapular inclination angle. The angle between the line connecting the center of the glenoid to the spine of the scapula and the vertical line on the midline of the vertebra is evaluated as the thoracoscapular angle. It is noteworthy that the scapular inclination angle and thoracoscapular angle together complement each other to form a total of 90 degrees (Figure 2).

**Figure 3:**
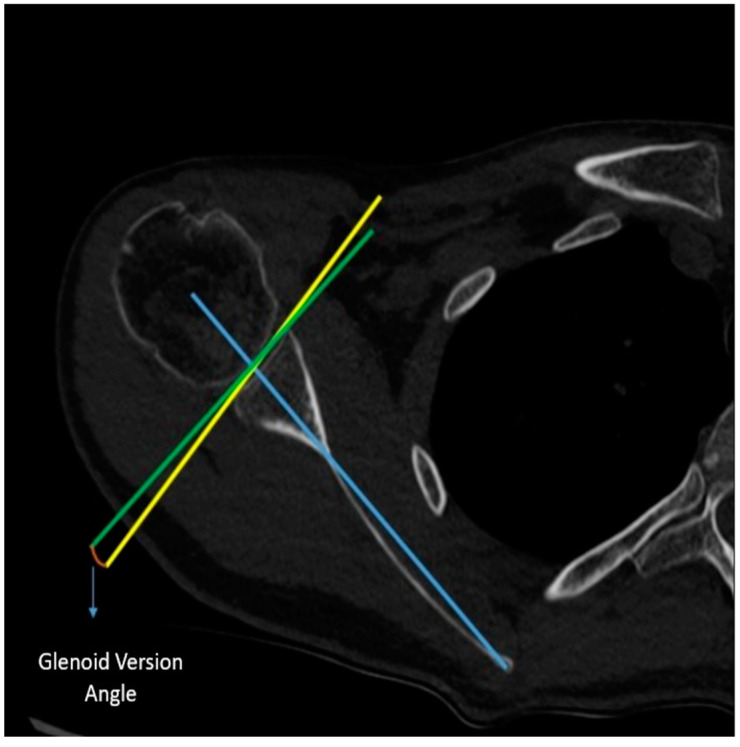
Measurement of the glenoid version angle.

Measurement of Glenoid Version Angle: In the axial CT imaging, a line (referred to as the Friedman line) is drawn from the most medial border of the scapula to the center of the glenoid fossa. Subsequently, a line perpendicular to this scapular axis is drawn through the center of the glenoid. Following that, a line connecting the anterior and posterior rims of the glenoid is drawn. The angle formed between the line drawn perpendicular to the scapular axis and the line connecting the corners of the glenoid is assessed as the glenoid version angle (Figure 3).

**Figure 4:**
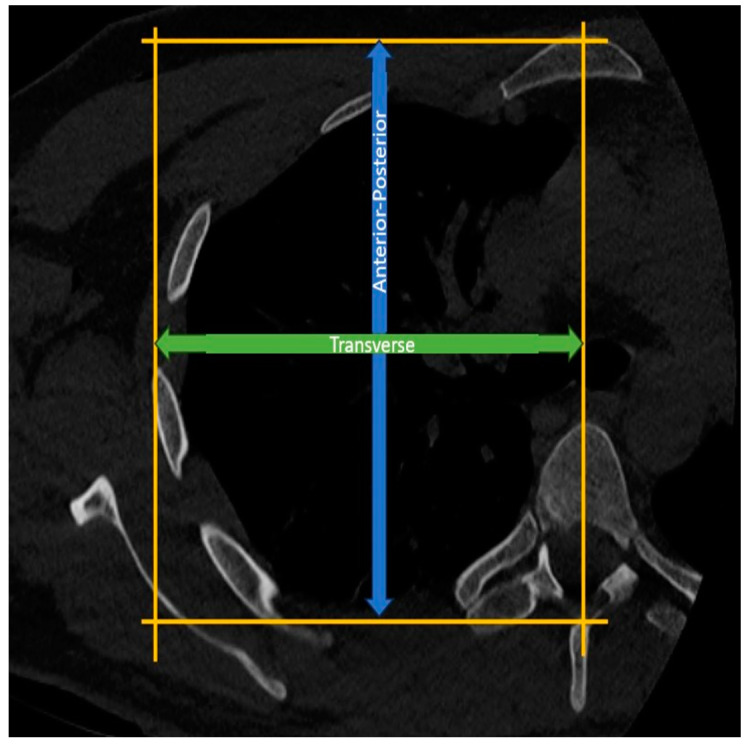
Measurement of anterior-posterior thoracic and transverse hemithoracic diameter.

Measurement of Thoracic Anterior-Posterior Diameter: In the axial CT sections at the T4-5 level, the length between a line drawn parallel to the sternum from the anterior surface of the sternum and another line drawn parallel to this line from the apex of the spinous process of the corresponding vertebra is assessed as the thoracic anterior-posterior diameter (Figure 4).

Measurement of Hemithoracic Transverse Diameter: In the axial CT sections at the T4-5 level, a vertical line is drawn from the midline of the vertebra, connecting a line drawn parallel to the sternum from the anterior surface of the sternum and another line drawn parallel to this line from the lateralmost part of the corresponding rib. The distance between these lines is then assessed as the thoracic transverse radius (Figure 4).

The measurements of thoracic diameter were conducted based on morphometric standards employed in the literature by disciplines such as thoracic surgery and pulmonology [6]. The reason for standardizing the method to the T4-5 level was also based on the level of the scapular spine.

### 2.3. Statistical Analysis

To thoroughly investigate the relationship between thoracic morphology and the alpha angle, a multivariate regression analysis was conducted, incorporating potential confounding variables such as age, gender, and body mass index (BMI). This approach allowed for a more nuanced understanding of the factors influencing the alpha angle. Additionally, inter-observer reliability for the radiological measurements was evaluated using the intraclass correlation coefficient (ICC), ensuring the robustness of the data.

Descriptive statistics were presented as means ± standard deviations or medians with interquartile ranges, depending on data distribution. The Shapiro-Wilk test was used to assess normality. Pearson and Spearman correlation coefficients were calculated for parametric and non-parametric data, respectively. For the AP/T ratio’s predictive capacity on alpha angle deviations, a receiver operating characteristic (ROC) curve analysis was performed, and sensitivity and specificity values were calculated. IBM SPSS v28.0 was utilized for all analyses, with *p* < 0.05 considered significant.

## 3. Results

Patients who underwent the Latarjet procedure for recurrent shoulder instability between 2022 and 2024 were studied in a single center by a single surgeon. Among the 74 patients, 90% (*n* = 67) were male, and 10% (*n* = 7) were female with a mean BMI of 24.3 ± 3.1 kg/m^2^. Their ages ranged from 16 to 45, with an average age of 26.4 ± 6.4 years. The surgical procedures were performed on the right side in 60% of the cases and on the left side in 40%. A significant relationship was noted between thoracic morphology and the alpha angle in postoperative CT scans. There was a significant correlation between the anterior-posterior/transverse diameter ratio (AP/T) and the alpha angle (r = 0.407, *p* < 0.001). A significant correlation was also found between scapular inclination and the alpha angle (r = 0.275, *p* = 0.018), as well as between the glenoid version and the alpha angle (r = 0.241, *p* = 0.039). As expected, a significant negative correlation was observed between the thoracoscapular angle and the alpha angle (r= −0.288, *p* = 0.013). The multivariate analysis revealed that the AP/T ratio was an independent predictor of alpha angle deviations, even after adjusting for age, gender, and BMI (β = 0.42, *p* < 0.001). Inter-observer reliability testing yielded an ICC of 0.88 (95% CI: 0.88–0.96), indicating excellent agreement.

**Figure 5:**
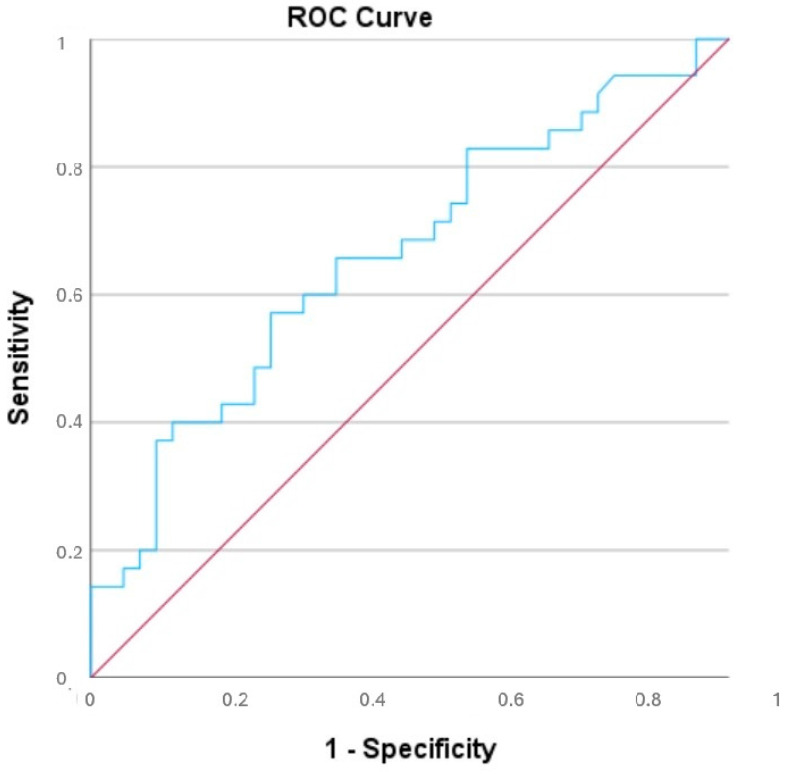
Logistic regression curve analysis showing the effect of AP/T on the alpha angle.

**Figure 6:**
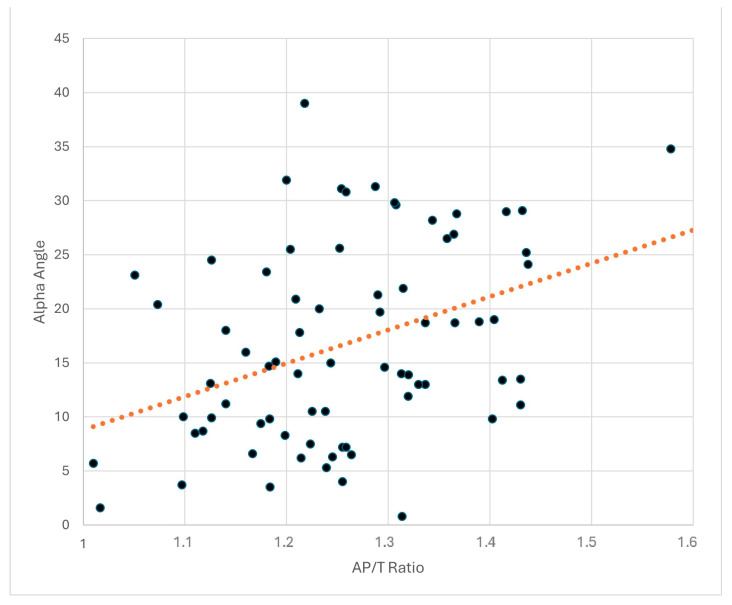
Scatter plot showing the relationship between alpha angle and AP/T ratio. The orange dotted line represents the linear trend, indicating a positive correlation between the variables.

An alpha angle value of 15 degrees was set as the threshold, and the patients were divided into two groups. Below the alpha angle of 15, there were 39 patients, while above 15, there were 35 patients. An alpha angle above 15 degrees was determined to be associated with poor outcomes. Descriptive statistical data of the two groups divided according to alpha angle are shown in Table 1. ROC analysis determined that an AP/T ratio threshold of 1.2545 had a sensitivity of 75% and specificity of 62% for predicting alpha angle deviations exceeding 15°. The corresponding area under the curve (AUC) was 0.660 (*p* = 0.018) (Figure 5). These findings suggest that the thoracic wall morphology directly influences the alpha angle. Scatter plots illustrating the relationships between thoracic morphology parameters and alpha angle are presented in Figure 6.

## 4. Discussion

To our knowledge, this is the first study to demonstrate a direct relationship between thoracic morphology and the alpha angle. Our findings indicate that the position of the scapula on the thoracic wall directly affects the placement of screws during surgery. In patients with a high AP/T diameter ratio, an increase in the alpha angle was observed, supporting our hypothesis.

Postoperative changes in scapular inclination and thoracoscapular angle following the Latarjet procedure have been shown in the literature [5]. Cerciello et al. observed a decrease in the thoracoscapular angle in the early postoperative period, indicating a more vertical positioning of the scapula on the thoracic wall. Despite the release of the pectoralis minor muscle, they hypothesized that this change was amplified by the coracobrachialis muscle’s protraction due to the coracoid block transfer. This reported change, measured at 4 degrees, was observed to normalize and become equal to the unaffected side by the 6th postoperative month. Our study considered chest CT scans taken in the early postoperative period, not accounting for these early changes in scapular inclination. Since the thoracic AP/Transverse diameter measurements were taken from bony landmarks, we believe they are not affected by postoperative changes. Future studies should include patients with non-standard thoracic anatomy or prior shoulder surgeries to assess whether these factors influence alpha angle variability.

The literature shows that the postoperative alpha angle directly affects the procedure’s outcome. Hsu [4] et al., in a cadaveric biomechanical study, examined the stability of the coracoid block transfer. Their findings revealed no significant difference at angles between 0 and 15 degrees, but stability markedly decreased under cyclic loadings in samples positioned at a 30-degree alpha angle. Other authors in the literature have also indicated 15 degrees as the stability limit [7]. In our study, taking 15 degrees as a limit was made in line with these studies.

Some suggestions have been made in the literature to reduce this angle. There are several studies in this context. Barth [8] et al. suggested using a drill guide in addition to the free-hand technique to reduce this angle. Tang [9] et al. demonstrated that using a longer drill guide could effectively reduce this angle. We find it useful to use one of these methods in patients with a high AP/T diameter ratio on preoperative CT evaluation. A comparative study between free-hand and guided screw techniques would provide further insights into optimizing surgical approaches to reduce alpha angle deviations.

In our study, a large portion of the sample (90%) consisted of male patients, and differences in male and female thoracic morphology were not taken into account. The existing literature suggests that the AP/Lateral diameter ratio is similar in both sexes [10]. Despite variations in thoracic volume ratios between the sexes, the proportional similarity of the AP/Lateral diameter is the reason for not assessing this distinction in our study.

Longitudinal follow-up on clinical outcomes was excluded due to the focus on immediate postoperative anatomical measurements and the complexity of correlating these with long-term functional outcomes. Studies that will examine long-term results and compare them to early postoperative changes may reveal the clinical correlation of the biomechanical potential that occurs with graft position.

Our study has at least four limitations. (1) Alpha Angle Threshold Validation: The use of 15 degrees as the critical threshold for the alpha angle is based on biomechanical studies [4]. However, clinical validation of this threshold, particularly in diverse populations, is required to confirm its practical relevance. (2) Sample Demographics: The study predominantly included male patients (90%). Given potential anatomical differences between sexes, future studies should examine thoracic morphology in a more balanced sample to assess the generalizability of the findings. (3) Postoperative Changes: Only early postoperative CT scans were evaluated, without accounting for long-term changes in scapular inclination or thoracoscapular angle. Longitudinal studies would provide better insight into how these factors evolve and impact surgical outcomes over time. (4) Limited Clinical Correlation: While this study establishes a correlation between thoracic morphology and alpha angle, its direct impact on clinical outcomes such as stability, pain, or range of motion was not evaluated.

In light of this study, we have at least four suggestions for future direction. (1) Longitudinal Studies: Conducting studies that follow patients over time to observe the relationship between thoracic morphology, alpha angle, and clinical outcomes (e.g., shoulder function, recurrence of instability) would enhance the understanding of these associations. (2) Technical Optimization: Investigating the efficacy of guided screw placement techniques, particularly in patients with high AP/T ratios, could help mitigate intraoperative challenges and improve outcomes. (3) Broader Populations: Future research should include patients with varied thoracic morphologies, as well as those with pre-existing conditions or prior surgeries, to explore how these factors influence alpha angle variability. (4) Validation of AP/T Cutoff Values: The cutoff value of “1.2545” for the AP/T ratio requires validation in larger, multicenter cohorts to confirm its predictive accuracy for alpha angle deviations.

## 5. Conclusions

Our study clearly demonstrates that thoracic morphology directly affects the alpha angle observed in chest CT scans following the Latarjet procedure. Specifically, in patients with a higher anterior-posterior to transverse thoracic diameter ratio (AP/T), there is a proportional increase in the alpha angle. Given that a deviation beyond a certain degree in the alpha angle during the coracoid process transfer directly influences the procedure’s outcome, a preoperative understanding of thoracic morphology becomes crucial. In preoperative evaluation, if the AP/T ratio is above 1.25 on the chest CT scan, the surgeon may have difficulty achieving the target alpha angle (≤15). This knowledge can be advantageous for the surgeon in anticipating and addressing intra-operative challenges. Specifically, measuring the AP/T ratio using standard CT imaging can provide valuable predictive information, allowing surgeons to anticipate potential difficulties in achieving the desired alpha angle. In cases where the AP/T ratio exceeds 1.25, as identified by the ROC analysis in this study, surgeons should consider employing adjunct techniques, such as guided screw placement, to mitigate the risk of deviations that could compromise outcomes.

Future research should validate these findings across broader populations and explore the long-term clinical implications of alpha angle variations. By incorporating thoracic morphology into the preoperative workflow, surgeons can improve precision in surgical planning, ultimately enhancing patient outcomes in the management of recurrent shoulder instability.

## Figures and Tables

**Table 1:** Descriptive statistical data of 2 groups according to alpha angle.

Alpha Angle	≤15	>15
Patients (*n*)	39	35
Gender (M/F)	36/3	31/4
Age	26.07 ± 5.90	26.80 ± 6.84
Side (Right/Left)	22/17	23/12
AP/T	1.22 ± 0.10	1.29 ± 0.12
Scapular Inclination	43.20 ± 7.06	46.46 ± 8.41
Thoracoscapular Angle	47.31 ± 7.04	43.42 ± 8.67
Glenoid Version	−1.57 ± 5.64	0.97 ± 4.78
Alpha Angle	9.46 ± 3.86	25.08 ± 5.42

## Data Availability

The data supporting the findings of this study are not publicly available due to privacy or ethical restrictions. For access to the study data, please contact the corresponding author at mfdarilmaz@gmail.com.

## List of Abbreviations

CT	Computed Tomography
PACS	Picture Archiving and Communication Systems
AP/T	Anterior-Posterior/Transverse Diameter Ratio

## References

[1] Latarjet M. (1954). Treatment of recurrent dislocation of the shoulder. Lyon Chir..

[2] Hovelius L., Sandström B., Olofsson A., Svensson O., Rahme H. (2012). The effect of capsular repair, bone block healing, and position on the results of the Bristow-Latarjet procedure (study III): Long-term follow-up in 319 shoulders. J. Shoulder Elb. Surg..

[3] Schmid S.L., Farshad M., Catanzaro S., Gerber C. (2012). The Latarjet procedure for the treatment of recurrence of anterior instability of the shoulder after operative repair: A retrospective case series of forty-nine consecutive patients. J. Bone Jt. Surg. Am..

[4] Hsu K.L., Yeh M.L., Kuan F.C., Hong C.K., Chuang H.C., Wang W.M., Su W.R. (2022). Biomechanical comparison between various screw fixation angles for Latarjet procedure: A cadaveric biomechanical study. J. Shoulder Elb. Surg..

[5] Cerciello S., Edwards T.B., Cerciello G., Walch G. (2015). Scapular position after the open Latarjet procedure: Results of a computed tomography scan study. J. Shoulder Elb. Surg..

[6] Sverzellati N., Colombi D., Randi G., Pavarani A., Silva M., Walsh S.L., Pistolesi M., Alfieri V., Chetta A., Vaccarezza M. (2013). Computed Tomography Measurement of Rib Cage Morphometry in Emphysema. PLoS ONE.

[7] Hovelius L., Sandström B., Saebö M. (2006). One hundred eighteen Bristow-Latarjet repairs for recurrent anterior dislocation of the shoulder prospectively followed for fifteen years: Study II-the evolution of dislocation arthropathy. J. Shoulder Elb. Surg..

[8] Barth J., Boutsiadis A., Neyton L., Lafosse L., Walch G. (2017). Can a drill guide improve the coracoid graft placement during the latarjet procedure?: A prospective comparative study with the freehand technique. Orthop. J. Sports Med..

[9] Tang R.L.E., Mackenzie S.P., Cuzmar Grimalt D., Young A.A., Niu R., Mattern O.J., Cass B. (2022). Optimising Screw Trajectory in the Bristow-Latarjet Procedure: A Simple Technique to Improve the Alpha Angle. J. Orthop. Surg. Tech..

[10] Bellemare F., Jeanneret A., Couture J. (2003). Sex differences in thoracic dimensions and configuration. Am. J. Respir. Crit. Care Med..

